# RETRACTION: Influence of Closed‐Incision Negative‐Pressure Eound Therapy on the Incidence of Surgical Site Wound Infection in Patients Undergoing Spine Surgery: A Meta‐Analysis

**DOI:** 10.1111/iwj.70167

**Published:** 2024-12-15

## Abstract

**RETRACTION:** LiuZ.‐S. , TianS.‐B. , “Influence of Closed‐Incision Negative‐Pressure Eound Therapy on the Incidence of Surgical Site Wound Infection in Patients Undergoing Spine Surgery: A Meta‐Analysis,” International Wound Journal 20, no. 10 (2023): e14317, 10.1111/iwj.14317.PMC1068153737518769

The above article, published online on 30 July 2023, in Wiley Online Library (http://onlinelibrary.wiley.com/), has been retracted by agreement between the journal Editor in Chief, Professor Keith Harding; and John Wiley & Sons Ltd. Following an investigation by the publisher, all parties have concluded that this article was accepted solely on the basis of a compromised peer review process. In addition, the journal received a report from a third party which indicated that one reference in this study was inappropriately included in the meta‐analysis. The editors have therefore decided to retract the article. The authors did not respond to our notice regarding the retraction.

**RETRACTION:** Z.‐S. Liu , S.‐B. Tian , “Influence of Closed‐Incision Negative‐Pressure Eound Therapy on the Incidence of Surgical Site Wound Infection in Patients Undergoing Spine Surgery: A Meta‐Analysis,” International Wound Journal 20, no. 10 (2023): e14317, 10.1111/iwj.14317.PMC1068153737518769

The above article, published online on 30 July 2023, in Wiley Online Library (http://onlinelibrary.wiley.com/), has been retracted by agreement between the journal Editor in Chief, Professor Keith Harding; and John Wiley & Sons Ltd. Following an investigation by the publisher, all parties have concluded that this article was accepted solely on the basis of a compromised peer review process. In addition, the journal received a report from a third party which indicated that one reference in this study was inappropriately included in the meta‐analysis. The editors have therefore decided to retract the article. The authors did not respond to our notice regarding the retraction.

